# ANP32B promotes colorectal cancer cell progression and reduces cell sensitivity to PRAP1 inhibitor through up-regulating HPF1

**DOI:** 10.1016/j.heliyon.2023.e23829

**Published:** 2023-12-16

**Authors:** Li-Li Yang, Meng Li, Wei Huang, Peng-Tao Ren, Qing-Hui Yan, Ying-Hao Hao

**Affiliations:** a Department of Radiology, Xingtai People's Hospital, Xingtai, Hebei, China; b The Third Department of General Surgery, The Second Hospital of Hebei Medical University, Shijiazhuang, Hebei, China

**Keywords:** ANP32B, Colorectal cancer, HPF1, PARP1 inhibitor

## Abstract

ANP32B, a member of the acidic leucine-rich nuclear phosphoprotein 32 family member B, is aberrantly expressed in various cancers, including colorectal cancer. However, the function and mechanism of action of ANP32B in colorectal cancer remain unclear. The present study therefore analyzed the expression of ANP32B and its activity in colorectal cancer patient samples and colorectal cancer cell lines. ANP32B expression was found to be significantly upregulated in colorectal cancer patient samples and cell lines. Upregulation of ANP32B enhanced colorectal cancer cell proliferation and migration, whereas downregulation of ANP32B suppressed colorectal cancer cell proliferation. RNA sequencing analysis of differentially expressed genes in ANP32B silenced colorectal cancer cells showed that histone PARylation factor 1 (HPF1), which protects against DNA damage by interacting with the anti-tumor target PARP1, was significantly downregulated. Luciferase promoter assays testing the regulatory association between ANP32B and HPF1 showed that ANP32B interacted with the HPF1 promoter. Analysis of colorectal cancer samples from The Cancer Genome Atlas showed that ANP32B and HPF1 expression were positively correlated, and recovery assays showed that ANP32B promoted colorectal cancer progression by up-regulating HPF1. Overexpression of ANP32B also reduced the sensitivity of colorectal cancer cells to PARP1 inhibitor, consistent with the oncogenic role of ANP32B. ANP32B may alter the sensitivity of colorectal cancer cells to PARP1 inhibitor via a mechanism associated with the HPF1 gene. In summary, these findings showed that ANP32B acted as a tumor promoter, potentiating both colorectal cancer malignancy and drug resistance. Targeting the ANP32B/HPF1 axis may have benefit for patients with colorectal cancer.

## Highlights

• ANP32B is highly expressed in colorectal cancer patient samples.
• ANP32B promotes the progression of colorectal cancer by up-regulating HPF1.
• ANP32B regulates the sensitivity of CRC cells of PARP1 inhibitor.
• HPF1 functions as an oncogene in CRC.

## Graphical abstract

**Figure:**
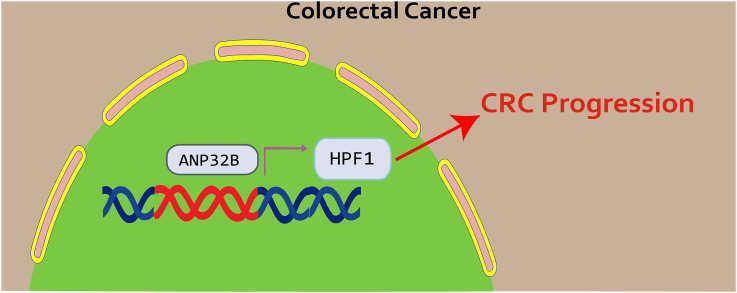

## 1 Introduction

Colorectal cancer (CRC) is the third most diagnosed malignancy worldwide, affecting more than 9.8 % of patients diagnosed with cancer, and the second leading cause of cancer-associated deaths, being responsible for 9.2 % of cancer deaths [1]. During the past years, The rates of CRC have increased worldwide [2] and in China [3]. The National Central Cancer Registry of China has estimated that, of patients diagnosed with cancer, approximately 3.8 per 1000 were newly diagnosed with CRC in 2015 and that, of patients who died of cancer, approximately 1.91 per 1000 died of CRC in 2015 [3]. Despite advances in CRC treatment, however, the 5-year overall survival (OS) rate remains 64.9 %, [4]. These findings suggest the need to identify novel biomarkers to predict the prognosis of patients with CRC and tumor drug sensitivity, which may help identify targets for CRC treatment.

The acidic leucine-rich nuclear phosphoprotein 32 family member is a family of regulatory proteins critical for intracellular transport, transcriptional regulation, protein phosphorylation, and cell-death pathways. The functions of the ANP32 proteins have been associated with their N-terminal leucine-rich repeat domains (LRRs) and their C-terminal low-complexity acidic regions (LCARs) [5]. To date, eight members of the ANP32 family have been identified in humans, with only three of these (ANP32A, ANP32B, and ANP32E) functioning in vertebrates [6]. ANP32s are dysregulated in human cancers, and their function in tumor development has shown context dependency. For example, ANP32A expression is reduced in prostate [7] and breast [8] cancers, but was increased in prostate cancer and CRC [9,10]. ANP32E has been identified as a negative prognostic marker in myeloma [11], but as a positive predictor of response to follicular lymphoma treatment [12].

*ANP32B*, also known as *PAL31*, was first cloned in 2000. The ANP32B protein was found to be located in the nucleus and be associated with brain development by regulating the proliferation of neuronal cells [13]. ANP32B was shown to be important for cell proliferation, apoptosis, and cell cycle progression [14,15], and to participate in various types of cancer, including leukemia [14,16], breast cancer [17,18], hepatocellular carcinoma [19,20], pancreatic adenocarcinoma [21] and thyroid carcinoma [22]. Although ANP32B expression was also found to be upregulated in patients with CRC [23], the precise function of ANP32B in CRC remains to be determined. To identify possible targets for CRC treatment, the present study investigated the expression and activity of ANP32B in CRC patients and cells.

## 2 Materials and methods

### 2.1 Samples from patients with colorectal cancer in The Cancer Genome Atlas

Datasets containing 471 CRC samples and 41 paracancerous samples were obtained from The Cancer Genome Atlas (Project ID, TCGA-COAD, https://portal.gdc.cancer.gov/projects/TCGA-COAD), and the levels of expression of ANP32B and histone PARylation factor 1 (HPF1) were analyzed. The correlation between ANP32B and HPF1 expression was determined by Pearson correlation analysis.

### 2.2 Cell culture and transfection

The human CRC cell lines RKO, HCT116 and HT29, and normal colorectal cells (NCM460) were obtained from the cell resource center of Peking Union Medical College (Beijing, China). Cells were maintained at 37 °C in RPMI-1640 medium containing 10 % fetal bovine serum (FBS; GIBCO, Carlsbad, CA) in a humidified atmosphere containing 5 % CO_2_.

Small interfering RNAs (siRNAs) targeting ANP32B (siANP32B#1, 5′- GCUUACCUACUUGGAUGGCUAdTdT-3’; and siANP32B#2, 5′- GAGGGCUUAACAGCUGAAUUUdTdT-3′) and HPF1 (siHPF1, 5′-GTGAAGAACTTGATCCTGAAA-3′) and a negative control (siCtrl, 5′- CAGUACUUUUGUGUAGUACAAA-3′) were designed and synthesized by Hippobio (Huzhou, China) for gene knockdown in RKO cells. For cell transfection, Invitrogen Biotechnology's Lipofectamine 3000 (Cat# L3000001, Thermo Fisher Scientific, Waltham, MA, USA) was used. The concentration of siRNAs used was 100 pmol for cell transfection in 6-well plates. Approximately, after transfected for 48–72 h, the cells were collected for further experiments. For gene overexpression in HCT116 cells, DNA fragments covering the ANP32B (NM_006401.2) and HPF1 (NM_017867.3) coding regions were amplified and cloned into pcDNA3.1 vector (Tianyihuiyuan), yielding pcDNA-ANP32B and pcDNA-HPF1, respectively. Blank vector was used as the negative control (Ctrl). Lipofectamine 3000 reagent (Promega) was used for cell transfection.

### 2.3 qRT-PCR

Total RNA was extracted from CRC cells using TRIzol Reagent (Invitrogen), followed by reverse transcription to cDNA using PrimeScript ™ RT reagent Kits and gDNA Erasers (Takara). qPCR reactions were performed with SYBR Premix Ex *Taq*II (Takara) on a Light Cycler Roche 480 II Real-Time PCR System (Roche, Basel Switzerland). Primers were designed to amplify sequences of ANP32B (forward, 5′- CTGTTCGAGAACTTGTCTTGGAC-3’; reverse, 5′- AGCTTGGGGAGATTTGAAACTG-3′). HPF1 (forward, 5′-TGGGGCTTGAATTGGGAATGG-3’; reverse, 5′- GCAGCAAGTTGGTCTATGTTCTC-3′) and β-actin (forward, 5′-CATGTACGTTGCTATCCAGGC-3′, reverse, 5′-CTCCTTAATGTCACGCACGAT-3′) mRNAs. Data were analyzed using the 2 ^–ΔΔCT^ method, with β-actin as the internal reference.

### 2.4 Western blotting

Total proteins were obtained from CRC cells, separated by 12 % SDS-PAGE, and transferred to PVDF membranes. The membranes were blocked by incubating in buffer containing 5 % skim milk and incubated overnight at 4 °C with primary antibodies against ANP32B (1:1000, GeneTex, Cat: GTX115437), HPF1 (1:1000; CUSABIO, Cat: CSB-EP865162HU) GAPDH (1:1000, proteintech, Cat: 60004-1-Ig) and β-actin (1:1000, SANTA, Cat: SC-47778). The membranes were washed and incubated for 1 h at 37 °C with secondary antibodies. Bands were visualized and quantitated using an Odessey CLx system (LI-GOR, USA).

### 2.5 CCK-8 assay

Cells viability was evaluated by the CCK-8 method (Beyotime, China). In brief, CRC cells transfected with siRNAs or plasmids were cultured for 24, 48, 72 and 96 h. To assess cell sensitivity to the PARP1 inhibitor AG-14361 (SC0029, Beyotime), CRC cells transfected with plasmids or treated with AG-14361 were cultured for 48 h, followed by the addition of 10 μl CCK-8 reagent and incubation for 2 h. The absorbance of each well at 450 nm was measured on a microplate reader (HBS-1096, DieTie, Nanjing, China).

### 2.6 Cell colony formation assay

CRC cells were treated with siRNAs, plasmids, and/or PARP1 inhibitor at various concentrations. Aliquots containing 5 × 10^3^ CRC cells were plated into each well of a six-well plate. After 14 days, the colonies were captured and incubated with a staining reagent containing formaldehyde, crystal violet, and methanol for 30 min. The cells were washed, air dried, photographed and counted using ImageJ software (NIH, Bethesda, MD).

For drug sensitivity assays, cells were divided into four groups and treated with pcDNA3.1 plasmid (NC group), pcDNA-ANP32B (OE group), pcDNA3.1 plasmid and 40 nM PARP1 inhibitor (NC + inhibitor group), pcDNA-ANP32B and 40 nM PARP1 inhibitor (OE + inhibitor group). The cell colonies were treated and counted as described above.

### 2.7 Transwell assays

Cell migration assays were performed by determining the number of cells migrating across transwell chambers. Briefly, transfected CRC cells (1 × 10^5^ cells/250 μl) in RPMI-1640 medium without PBS were added to the upper chamber of each well of a 24-well plate (Corning), and 650 μl of complete medium containing 20 % PBS was added to each lower chamber. The plates were incubated for 18–24 h and the migrating CRC cells were fixed with 70 % ethanol. After staining with 0.25 % crystal violet, the cells were counted using an inverted microscope (XD; Ningbo Sunny Instruments Co., Ltd., China).

### 2.8 RNA sequencing

RKO cells (8 × 10^4^) transfected with siANP32B (100 pmol) or siCtrl (100 pmol) and suspended in RPMI 1640 medium containing 10 % FBS was added to each well of a 6-well plate and cultured. Total RNA was extracted from each sample using TRIzol Reagent (Invitrogen), with the concentration and purity of the RNA samples determined by Nano-200 (ALLSHENG, Hangzhou, China). The total RNA samples were sent to Allwegene Corporation for cDNA library construction and RNA sequencing on Illumina HiSeq 4000 (Illumina) platform with paired-end sequencing readings.

### 2.9 Luciferase reporter assay

The sequence 1 kb up-stream of the HPF1 gene and containing the HPF promoter was synthesized by Tianyihuiyuan (Beijing, China) and sub-cloned into the pGL 3.1 luciferase reporter vector (Promega). RKO cells were co-transfected with pGL 3.1-HPF1, pRL-TK vector, ANP32B siRNA or ANP32B overexpressing construct (pcDNA-ANP32B) using lipofectamine 2000. After 24 h, the cells were harvested and analyzed using the Dual-Luciferase Reporter Assay System (Promega) according to the manufacturer's instructions [24].

### 2.10 Statistical analysis

All data were expressed as the mean ± S.D. Student's test was used to assess the differences between groups, while one-way ANOVA followed by Tukey's honestly significant difference post-hoc test among three or more groups. Statistical analysis was performed using GraphPad prism v8.0 software (GraphPad Software Inc., San Diego, CA, USA). P < 0.05 was classified statistically significant.

## 3 Results

### 3.1 ANP32B is highly expressed in colorectal cancer tissues and cells

**Fig. 1:**
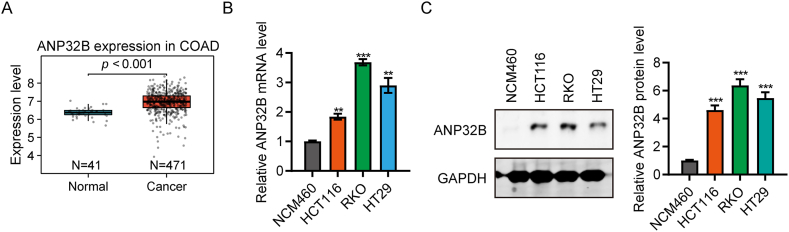
Expression of ANP32B in colorectal cancer tissue and cells. (A) Comparison of ANP32B expression in CRC tissues and paracancerous tissues from the TCGA database, showing that ANP32B expression was higher in CRC tissue samples. (B, C) Expression of (B) ANP32B mRNA, as shown by qPCR assays, and (C) ANP32B protein, as shown by western blotting, in three colorectal cancer cell lines (RKO, HCT116 and HT29) and normal colorectal cells (NCM460), showing that ANP32B mRNA and protein levels were higher in the three CRC cell lines. n = 3, **P < 0.01; ***P < 0.001.

Analysis of samples in the TCGA database showed that ANP32B expression was significantly higher in the 471 CRC samples than in the 41 paracancerous samples (Fig. 1A). Analysis of ANP32B RNA and protein levels in three CRC cells (HCT116, RKO and HT29) and a human normal colonic epithelial cell (NCM460) by qRT-PCR and western blotting, respectively, showed that the levels of expression of ANP32B mRNA (Fig. 1B) and protein (Fig. 1C) were higher in HCT116, RKO and HT29 cells than in NCM460 cells, suggesting that ANP32B may be a potential oncogene in CRC.

### 3.2 ANP32B promotes the proliferation and migration of colorectal cancer cells

**Fig. 2:**
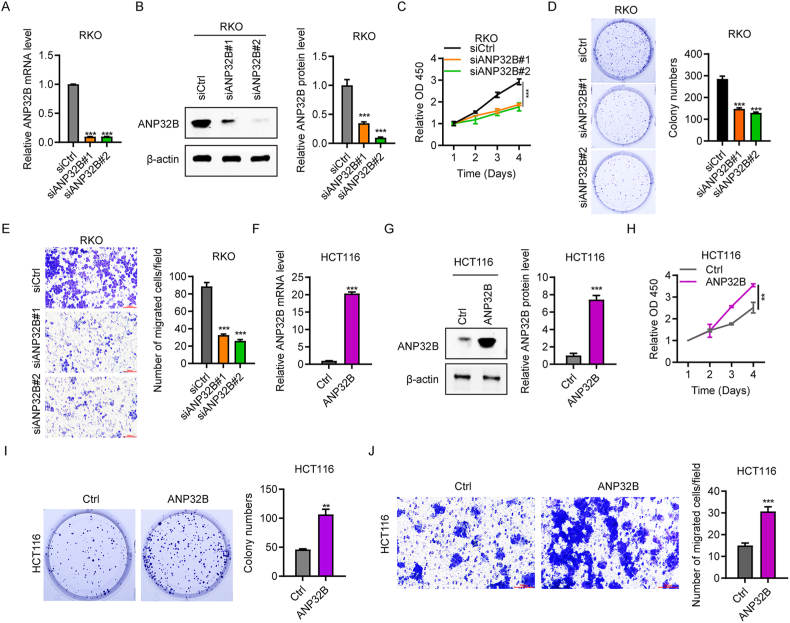
ANP32B promotes the amplification and migration of colorectal cancer cells. (A, B) Efficacy of ANP32B knockdown in RKO cells, as shown by (A) qPCR and (B) western blotting assays. n = 3. (C) Viability of RKO cells treated with siANP32B or siCtrl, as determined by CCK-8 assays. n = 3. (D) Numbers of colonies of RKO cells following treatment with siANP32B or siCtrl, as determined by cell colony formation assays. n = 3, **P < 0.01. (E) Migration of RKO cells transfected with siANP32B or siCtrl, shown by Transwell assays. n = 3, ***P < 0.001. (F, G) Confirmation of the overexpression of ANP32B mRNA and protein, as shown by (F) RT-qPCR and (G) western blotting, respectively. n = 3, ***P < 0.001. (H) Viability of HCT116 cells treated with pcDNA-ANP32B or Ctrl, as determined by CCK-8 assays. n = 3, **P < 0.01. (I) Numbers of colonies of HCT116 cells following treatment with pcDNA-ANP32B or Ctrl, as determined by cell colony formation assays. n = 3, **P < 0.01. (J) Migration of HCT116 cells treated with pcDNA-ANP32B or Ctrl, as determined by Transwell assays. n = 3, *P < 0.05.

To investigate the function of ANP32B in CRC progression, ANP32B was overexpressed in HCT116 cells or silenced in RKO cells, and cell viability, colony formation and cell migration were analyzed. RT-qPCR and western blotting confirmed that the expression of ANP32B was significantly lower in RKO cells transfected with siANP32B than with siCtrl (Fig. 2A and B). The CCK-8 assay showed that the growth rate of RKO cells was lower after ANP32B knockdown than with siCtrl (Fig. 2C). Cell colony formation and Transwell assays yielded similar results, with ANP32B knockdown suppressing cell colony formation (Fig. 2D) and cell migration (Fig. 2E).

Overexpression of ANP32B enhanced CRC cell growth and migration. RT-qPCR and western blotting confirmed that transfection of pcDNA-ANP32B significantly increased the expression of ANP32B in HCT116 cells (Fig. 2F and G). The CCK-8 assay revealed that the growth rate of HCT116 cells overexpressing ANP32B was higher than that of cells transfected with Ctrl vector (Fig. 2H). Moreover, ANP32B overexpression greatly enhanced cell colony formation and cell migration (Fig. 2I and J). Collectively, these results indicated that ANP32B effectively promotes the proliferation and migration of CRC cells.

### 3.3 ANP32B positively regulates the expression of HPF1

**Fig. 3:**
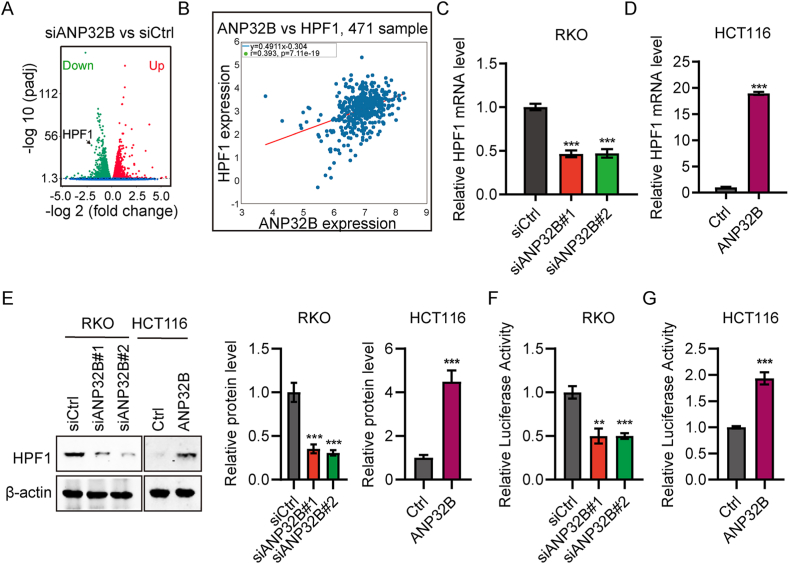
ANP32B positively regulates the expression of HPF1. (A) Volcano plots of genes differentially expressed by RKO cells transfected with siANP32B or siCtrl. The x-axis shows log2 fold changes and the y-axis shows the –log10 of corrective p value (q-value). (B) Correlation between ANP32B and HPF1 expression in patients with CRC, as determined by Pearson correlation coefficients. (C, D) RT-qPCR assays of HPF1 mRNA expression in RKO cells following (C) ANP32B knockdown or (D) ANP32B overexpression. n = 3, ***P < 0.001. (E) Western blot assays of HPF1 protein expression in cells following ANP32B overexpression or knockdown. (F, G) Luciferase reporter assays, showing the effects of ANP32B on HPF1 promoter activity after (F) ANP32B knockdown in RKO cells or (G) ANP32B overexpression in HCT116 cells. n = 3, **P < 0.01; ***P＜0.001.

Genes regulated by ANP32B within CRC cells were identified by RNA-Seq analysis using RKO cells transfected with siANP32B or siCtrl. A volcano plot identified the *HPF1* gene, which encodes histone PARylation factor 1, as being significantly downregulated after ANP32B knockdown (Fig. 3A). Analysis of the levels of expression of ANP32B and HPF1 in samples in the TCGA database showed a significant positive correlation (Fig. 3B). RT-qPCR and western blotting assays showed that both the mRNA and protein levels of HPF1 were positively regulated by ANP32B (Fig. 3C–E), and luciferase reporter assays showed that ANP32B promoted the transcription activity of HPF1 promoter (Fig. 3F and G). Taken together, these results suggested that ANP32B promotes HPF1 expression in CRC patients and cells.

### 3.4 ANP32B contributes to the migration and proliferation of CRC cells through upregulation of HPF1

**Fig. 4:**
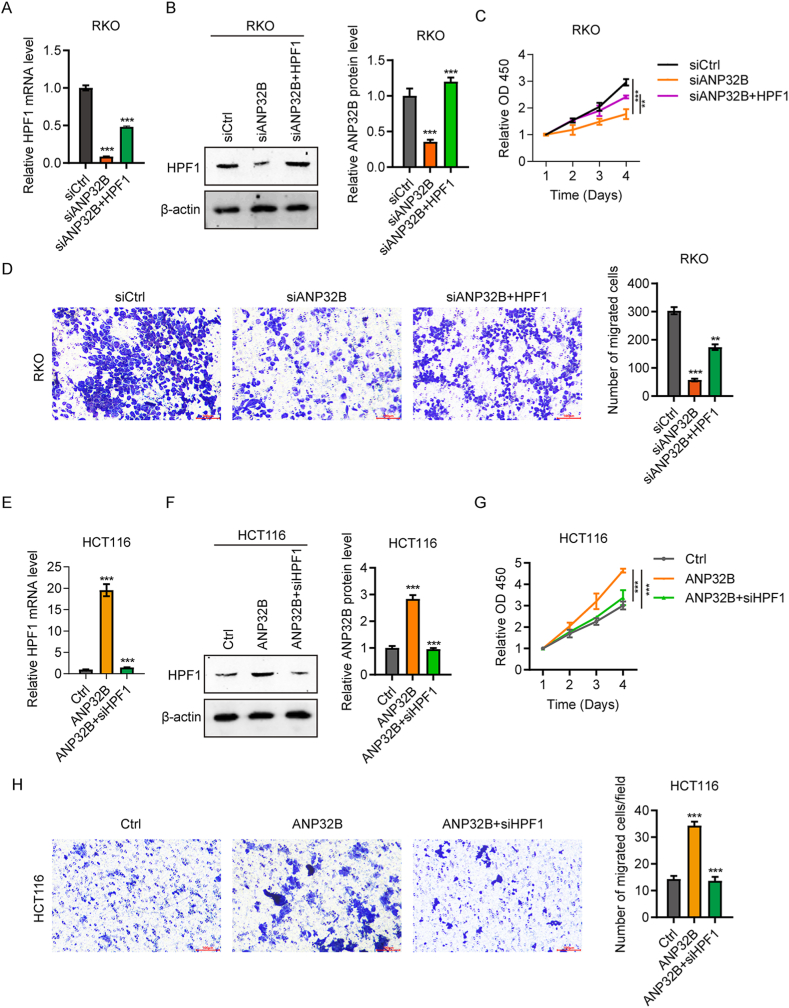
HPF1 contributes to the oncogenic effect of ANP32B on the amplification and migration of CRC cells. (A, B) Expression of (A) HPF1 mRNA expression as determined by RT-qPCR and (B) HPF1 protein as determined by western blotting in RKO cells transfected with siCtrl, siANP32B or siANP32B + HPF1. n = 3, ***P < 0.001. (C) CCK8 assays of the proliferation of RKO cells transfected with siCtrl, siANP32B or siANP32B + HPF1. (D) Transwell assays showing the migration of RKO cells transfected with siCtrl, siANP32B and siANP32B + HPF1. n = 3, **P < 0.01; ***P＜0.001. (E, F) Expression of HPF1 mRNA expression as determined by RT-qPCR (E) and (F) HPF1 protein as determined by western blotting in HCT116 cells in the Ctrl, ANP32B and ANP32B + siHPF1 groups. n = 3, ***P < 0.001. (G) CCK8 assays of the proliferation of HCT116 cells in the Ctrl, ANP32B and ANP32B + siHPF1 groups. (H) Transwell assays showing the migration of HCT116 cells in the Ctrl, ANP32B and ANP32B + siHPF1 groups. n = 3, *P < 0.05; **P < 0.01.

To further investigate the relationship between HPF1 and ANP32B in CRC cells, HPF1 was ectopically expressed in ANP32B knockdown RKO cells, followed by examination of cell proliferation and migration. ANP32B knockdown reduced the growth of RKO cells and the expression of HPF1, whereas pcDNA-HPF1 transfection into ANP32B knockdown RKO cells increased HPF1 expression (Fig. 4A and B) and their growth ability (Fig. 4C). Similar results were observed when cell migration was assessed. ANP32B knockdown reduced the ability of RKO cells to migrate, a reduction rescued by HPF1 overexpression (Fig. 4D).

Analysis of the effects of ANP32B overexpression and HPF1 knockdown on HPF1 levels in HCT116 cells showed that the HPF1 levels were increased following ANP32B overexpression and decreased after siHPF1 treatment (Fig. 4E and F). HPF1 inhibition reduced the viability of ANP32B overexpressing HCT116 cells to a normal level (Fig. 4G), as well as reducing the migration ability of HCT116 cells overexpressing ANP32B (Fig. 4H). Taken together, these results indicate that HPF1 expression restores the effects of ANP32B on the amplification and migration of CRC cells.

### 3.5 ANP32B expression regulates the sensitivity of CRC cells to PARP1 inhibitor

**Fig. 5:**
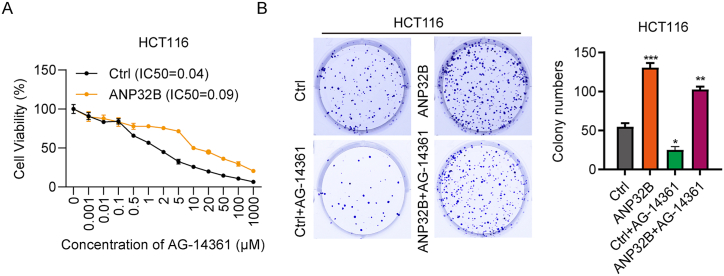
ANP32B expression reduces HCT116 cell sensitivity to the PARP1 inhibitor AG-14361. (A) Viability and (B) colony formation of HCT116 cells overexpressing ANP32B after 48 h of treatment with AG-14361. n = 3, *P < 0.05; **P < 0.01; ***P < 0.001.

Because HPF1 is a DNA protective factor that limits the hyper auto-modification of PARP1 [25], the effect of ANP32B expression on CRC cell sensitivity to PARP1 inhibitor was analyzed. Treatment of HCT116 cells that did and did not overexpress ANP32B with the PARP inhibitor AG-14361 [26] showed that this inhibitor enhanced the viability of cells overexpressing ANP32B but not control HCT116 cells (Fig. 5A). Similar results were observed when cell colony formation was assessed. PARP1 inhibitor significantly reduced the number of colonies of control HCT116 cells, but this effect was overcome by ANP32B overexpression (Fig. 5B). These results reveal that ANP32B overexpression is responsible for the resistance of CRC cells to PARP1 inhibitors.

## 4 Discussion

Dysregulation of ANP32B has been shown to participate in the development of human cancers. For example, ANP32B is highly expressed in patients diagnosed with chronic myelogenous leukemia (CML), whereas deletion of ANP32B enhances p53 transcriptional activity and synergizes therapeutically with tyrosine kinase inhibitors to inhibit CML propagation [16]. In contrast, however, ANP32B has been reported to activate caspase-3 to promote the apoptosis of leukemia cells [15]. Higher ANP32B mRNA expression is a marker of poor prognosis in patients with breast cancer [17], whereas ANP32B was found to suppress breast cancer tumor growth [18]. ANP32B mRNA and protein are highly expressed in hepatocellular carcinoma (HCC), with these levels of expression associated with tumor cell proliferation and invasion, suggesting that ANP32B is pro-oncogenic in HCC [19]. In contrast, ANP32B downregulation was found to have antiapoptotic effects in HCC, whereas ANP32B upregulation did not lead to HCC apoptosis and functioned as a cell cycle progression factor in HCC [20].

The present study was designed to determine the level of expression and function of ANP32B in CRC. ANP32B was found to be abundantly expressed in CRC cells. Upregulation of ANP32B promoted CRC cell amplification and migration, whereas inhibition of ANP32B suppressed CRC cell progression, indicating that ANP32B functions as an oncogene in CRC.

To determine the underlying mechanism by which ANP32B triggers CRC progression, RKO cells transfected with siANP32B or siCtrl were subjected to RNA-seq analysis to identify differentially expressed genes (DEGs). A PARP1 related gene, HPF1 (also called C4orf27), which encodes histone PARylation factor 1, was found to be one of the genes was found to be one of the genes markedly downregulated in ANP32B-silenced cells. RT-qPCR and Western blot assays confirmed that both HPF1 mRNA and protein levels were regulated by ANP32B. Luciferase reporter assays showed that ANP32B activated the transcription of HPF1 gene, and a positive correlation between ANP32B and HPF1 expression was observed in CRC patients. Taken together, these results confirmed that ANP32B stimulates the expression of HPF1 in CRC tissues and cells.

HPF1 is an accessory factor interacting with PARP1/2, which is specific for DNA damage response, indicating that HPF1 plays a vital role in response to PARP inhibitors [27]. PARP1 is a poly ADP-ribose polymerase enzyme that acts as a master regulator of DNA damage responses. Inhibitors of PARP1 are widely used clinically in the treatment of cancers [28], including CRC [29,30]. Nearly all patients with CRC develop drug resistance, limiting the therapeutic efficacy of anticancer agents [31]. The present study hypothesized that ANP32B might regulate the sensitivity of CRC cells to PARP1 inhibitors through HPF1. Evaluation of the effects of ANP32B on cell sensitivity to the PARP1 inhibitor AG-14361 showed that ANP32B upregulation suppressed CRC cell sensitivity to PARP inhibitors.

The present study still has some limitations. First, we only explored the effect of ANP32B/HPF1 axis on malignant phenotype of glioma from in vitro experiments, without further verification in vivo. In addition, the molecular mechanisms of ANP32B mediated regulation of HPF1 expression in CRC also requires further investigation.

## 5 Conclusion

In conclusion, the present study showed that the ANP32B/HPF1 axis contributed to regulating the progression of CRC. ANP32B overexpression may reduce CRC cell sensitivity to PARP1 inhibitor by upregulating HPF1. These findings may assist in developing preventive strategies that enhance CRC cell resistance to PARP1 inhibitors.

## Funding statement

This study was supported by Hebei medical science research project plan (NO. 20211335).

## Data availability statement

RNA sequencing data that support the findings of this study have been deposited in the Gene Expression Omnibus under accession code GSE247230. The datasets used and analyzed during the current study are available from the corresponding author on reasonable request.

## CRediT authorship contribution statement

**Li-Li Yang:** Writing - original draft, Validation, Methodology, Investigation. **Meng Li:** Writing - review & editing, Validation, Investigation, Formal analysis. **Wei Huang:** Writing - review & editing, Validation, Methodology, Formal analysis. **Peng-Tao Ren:** Writing - review & editing, Validation, Methodology, Formal analysis. **Qing-Hui Yan:** Writing - review & editing, Software, Formal analysis, Data curation. **Ying-Hao Hao:** Writing - original draft, Supervision, Funding acquisition, Conceptualization.

## Declaration of competing interest

The authors declare that they have no known competing financial interests or personal relationships that could have appeared to influence the work reported in this paper.

## Figures and Tables

**Figure:**
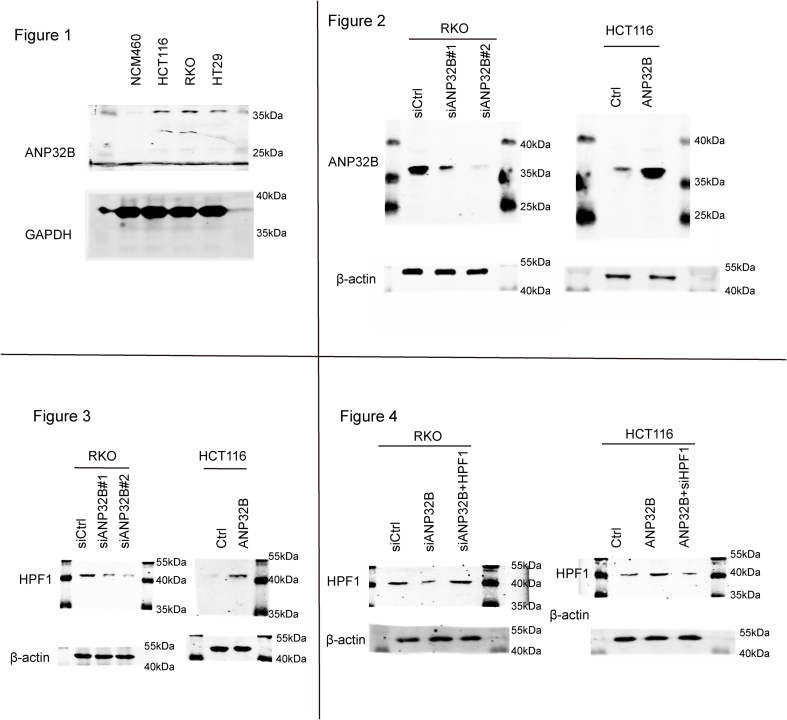


## List of abbreviations

CRC: colorectal cancer
HPF1: histone PARylation factor 1
TCGA: The Cancer Genome Atlas
OS: overall survival
HCC: hepatocellular carcinoma
*qRT-PCR*: quantitative real-time polymerase chain reaction
